# The Primate Cultural Significance Index: applications with Popoluca Indigenous people at Los Tuxtlas Biosphere Reserve

**DOI:** 10.1186/s13002-021-00483-8

**Published:** 2021-10-09

**Authors:** Marianna Pinto-Marroquin, John F. Aristizabal, Yasminda García-Del Valle, Felipe Ruan-Soto, Juan Carlos Serio-Silva

**Affiliations:** 1 Instituto de Ecología A.C, Carretera antigua a Coatepec No. 351, El Haya, CP 91070 Xalapa, Veracruz Mexico; 2 Grupo de Estudios Transdisciplinarios en Primatología, Red de Biología y Conservación de Vertebrados, Instituto de Ecología AC, Carretera antigua a Coatepec No. 351, El Haya, CP 91070 Xalapa, Veracruz Mexico; 3 Departamento de Ciencias Químico Biológicas, Instituto de Ciencias Biomédicas, Universidad Autónoma de Ciudad Juárez, Ciudad Juárez, Av. Benjamin Franklin No. 4650, Zona Pronaf, CP. 32310 Cd. Juárez, Chihuahua Mexico; 4 Laboratorio-Taller de Procesos Bioculturales, Educación y Sustentabilidad. Instituto de Ciencias Biológicas, Universidad de Ciencias y Artes de Chiapas, Libramiento Norte Poniente No. 1150. Colonia Lajas Maciel, C.P. 29039 Tuxtla Gutiérrez, Chiapas Mexico; 5 Instituto de Ciencias Biológicas, Universidad de Ciencias y Artes de Chiapas, Libramiento Norte Poniente No. 1150. Colonia Lajas Maciel, C.P. 29039 Tuxtla Gutiérrez, Chiapas Mexico

**Keywords:** Ethnoprimatology, Primates, Popoluca Indigenous people, Natural protected areas, Cultural significance, Conservation, Los Tuxtlas, Mexico

## Abstract

**Background:**

The study of the cultural significance (CS) of biodiversity provides key information to develop conservation strategies consistent with traditions and perceptions of human communities. In Los Tuxtlas Biosphere Reserve (TBR) in Mexico, the mantled howler monkeys (*Alouatta palliata mexicana*) and the black-handed spider monkeys (*Ateles geoffroyi vellerosus*) have historically coexisted with Popoluca Indigenous Peoples. This study sought to determine how the presence of a natural protected area (TBR location) and a range of sociodemographic factors (gender, age, origin, language proficiency, education level, religion) relate to the CS held by the Popoluca Indigenous People in relation to these two endangered primate species.

**Methods:**

The first Primate Cultural Significance Index (PCSI) was designed as a composed index of 11 cultural variables (sub-indices) and was applied randomly to a representative size sample of people over 15 years old in two Popolucas communities, one within the TBR (Piedra Labrada = 81 people) and another outside (Los Mangos = 91). U Mann–Whitney tests were used to compare the PCSI between communities and Generalized Linear Models (GLM) to evaluate the sociodemographic factors of participants that influenced the sub-indices in the PCSI.

**Results:**

The cultural significance of spider monkeys held by the Popolucas was higher for the community within the TBR than for the community outside, while for howler monkeys it was higher outside. For both primate species across the two communities, the most relevant sub-indices were (1) interest in conservation and (2) touristic significance of primates. Sociodemographic factors of participants influenced nine sub-indices of cultural significance out of the possible 10 sub-indices applied for each primate species. The demographic factors that most influenced each sub-index for both species were location and gender*.*

**Conclusions:**

The main differences found between communities may be linked to the conservation and sustainable development programs promoted by the reserve, as well as the greater persistence of Popolucan ancestral traditions within the boundaries of the reserve. We recommend that conservation efforts should focus on people less interested about primate conservation (women, non-natives and residents outside the reserve), and turn to the leadership of people more interested (native men who reside inside the reserve).

**Supplementary Information:**

The online version contains supplementary material available at 10.1186/s13002-021-00483-8.

## Background

Currently, it is difficult to find wild primate populations without some human influence [1]. For this reason, it is critical to study interactions between nonhuman primates (hereafter primates) and people [2]. Those interactions and their effect on animal conservation are the main object of study in ethnoprimatology [3]. Ethnobiological studies show that primates play a significant cultural role in many societies around the world [4, 5]. In Southeast Asia, primates are sold for the pet trade or to be consumed as medicine. They are also given important symbolism; are seen as holders of moral principles and their protection is associated with the sacred sites they habit [6–8]. Throughout Mesoamerica and South America, primates have also been used for a range purposes, including as food, medicine, and as raw materials to make cultural artifacts. In Mesoamerica, primates are sold by local people into the wildlife trade in order to resolve economic needs [9–15]. Likewise, primates are important in the symbolism and cosmovision of several ancestral cultures of the Neotropics [10, 16–19].

The value and role that any species plays in human cultures is defined as Cultural Significance – CS [20]. Culturally significant species are defined as those used as food or medicine, playing a role in religious practices or group mythology, or that have negative connotations (e.g., poisonous, invasive) [21]. To quantify the role of any organism in a particular culture, Turner (1988) proposed the Cultural Significance Index (CSI). He claimed that, despite the limitations that this index could have, it is less subjective and allows researchers to quantify cultural significance in a systematic and impartial way [21]. Nevertheless, the success of using this kind of index depends on the clarity and accuracy of its measurement, and therefore it is critical to utilize solid indicators or variables as well as to have a deep knowledge of the study context [20, 22].

Since the development and use of Turner’s first CSI, the cultural significance of organisms has been measured using indices in different ways. One of the simplest, common and most accurate of these is the Frequency of Mention Index [23, 24], which has been applied successfully to plants and fungi. CS has also been measured by using composed indices, considering value of use, cultural consensus, and data aggregation, among others [22, 25, 26]. Currently, there are no CSI designed and applied for primates only, but some are in use for vertebrates in general [27]. Studies to date have shown that the presence of natural protected areas and various social and demographic factors, such as gender, age, education level and religion, can influence perceptions and cultural values about biodiversity [28–30].

On the American continent, Los Tuxtlas region in southern Mexico represents the boreal limit of the tropical forest [31] and the most northern habitat for two of the three primate species present in Mexico, the mantled howler monkey (*Alouatta palliata mexicana*) and the spider monkey (*Ateles geoffroyi vellerosus*) [32, 33]. These primates have historically coexisted with the Popoluca Indigenous Peoples, the native ethnic group of Los Tuxtlas region [34, 35]. Traditionally, the Popolucas have used the natural resources guided by their traditions, satisfying their livelihood needs without negatively affecting the ecosystems [36]. However, these traditions are at risk of disappearing due to biodiversity loss, and recent socio-economic and demographic conditions [36, 37].

The original forest cover in Los Tuxtlas region, which was once 88%, has been lost principally as a result of cattle farming and agriculture [38]. At present, 60% of the landcover is for human use and the rest consists of fragmented forest [39]. Since 1937, several different conservation programs have been established in the region, but it was not until 1998 that 155.122 ha of the area was declared as Biosphere Reserve [36]. The aim of Biosphere Reserves of UNESCO is to harmonize interactions between people and nature for conservation and sustainable development, taking concerns and community participation into account in the management of protected areas [40]. In Los Tuxtlas Biosphere Reserve, conservation policies have thus sought to balance biodiversity conservation with local development, creating opportunities for human communities [41]. Around the world it is increasingly recognized that to achieve greater effectiveness in management and conservation of protected natural areas, local perception and cultural values must be considered [42]. With regard to primates, the study of the cultural significance provides key information to develop conservation strategies consistent with traditions and perceptions of people who share territories with these species [43].

This study aimed to: (1) measure and compare the cultural significance (CS) for two endangered primate species (*A. geoffroyi* and *A. palliata*) between two Indigenous Popoluca communities (one within Los Tuxtlas Biosphere Reserve (TBR), and another out of it); (2) evaluate the possible importance of social and demographic factors (gender, age, origin, perceived language proficiency, education level, occupation, and location) on the cultural significance index (CSI) of primates in the two Popolucan communities. In general, Biosphere Reserves seek to incorporate the local communities concerns and participation into management. For Popolucas, the TBR represents an opportunity for their traditional resource management to be integrated. Thus, we hypothesized that the CSI primates will play a more significant cultural role within the Popoluca community within the TBR than outside.

## Methods

### Study area and human communities

**Fig. 1:**
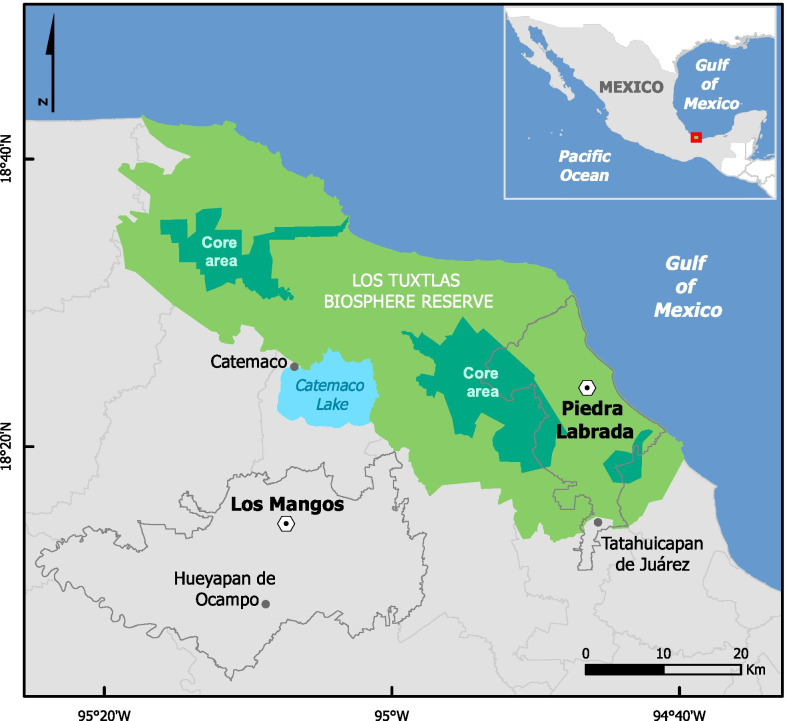
Study area and communities inside and outside of Los Tuxtlas Biosphere Reserve (TBR), Veracruz, Mexico. Piedra Labrada = Community inside the TBR; Los Mangos = Community outside the TBR

This study was conducted in 2017 in two Popoluca Indigenous communities in the state of Veracruz, Mexico: Piedra Labrada (inside the TBR) and Los Mangos (outside the TBR), (Fig. 1). The TBR is at the limit of the northern distribution of the tropical rainforest on the American continent [31] with an altitude ranging from 0 to 1780 masl and covering an area of 155,122 ha [44]. The climate is warm and humid (mean annual temperature = 25 °C, mean annual precipitation = 4 900 mm) [45] and the vegetation type is evergreen rainforest [46].

The community of Piedra Labrada (18°23′40" N, 94°46′35" W) is located inside the TBR, in the management zone designated for “Sustainable Use of Ecosystems”, which is a part of the buffer zone. Piedra Labrada has a population of 510 inhabitants of which 56% are self-identified Indigenous [47]. The community of Los Mangos (18°13′32" N, 95°08′22" W), located 16 km outside the reserve, has a population of 4131 inhabitants of which 16% are self-identified as Indigenous [47].

The native ethnic group of Los Tuxtlas is the Popoluca. They consider themselves as the children of *Homshuk*, the god of corn [34]. The Popolucas’ native language, Popolucan, is spoken in their communities and many pre-hispanic traditions are maintained, some of which are related to the management of natural resources [35]. However, due to the economic changes and growing demographic pressures from outside populations, as well as the influence of the Catholic and Protestant churches, these traditions are currently at risk [35, 36].

### Sequential study design

In this study, we adhered to the code of ethics by the Latin American Society of Ethnobiology [48]. The research consisted in four sequential phases: (1) Preliminary visit: the project was presented and the permission to conduct the research was requested of local leaders and authorities. (2) Period of qualitative information: semi-structured interviews were conducted with environmental and traditional leaders, elders and people living close to monkeys, obtaining information on their perceptions and cultural aspects regarding human-primate interactions such as the role of primates in the myths and legends, rituals, popular beliefs and archaeological representations, as well as their use at a medicinal, nutritional and economic level, and as pets. Those qualitative aspects were published in Pinto-Marroquin and Serio-Silva (2020) [49]. (3) Questionnaire design: based on the information obtained during the previous phase, variables reflecting the cultural importance of primates were identified and the Primate Cultural Significance Index (PCSI) and its questionnaire were designed to quantitatively assess the cultural significance (Additional file 1). The Edible Mushrooms Cultural Significance Index (EMCSI) [22, 50] and the Cultural Food Significance Index (CFSI) [51] were adapted for this study. A pilot survey was conducted with 14 men and 14 women from the Popoluca population. (4) Data collection: the permission to survey and the consent to publish responses from each person were granted before conducting the study. The questionnaire consisted of 22 closed multiple choice questions regarding cultural variables (sub-indices). The first question inquired about which primates were present in the locality, and photographs of *A. geoffroyi* and *A. palliata* were shown to verify their identity. Regardless if one of the species was mentioned or not, all of the same questions were asked for both species. One last question measured the importance of Los Tuxtlas Biosphere Reserve and was not included when calculating PCSI. The sample size (how many people were surveyed) for each community was calculated on the survey system web page (http://www.surveysystem.com/sscalc.htm#one) using a confidence level of 95% and a confidence interval of 10. The sample size for the total population over 15 years old inside the TBR (Piedra Labrada = 356 inhabitants) was 76 and 93 outside (Los Mangos = 2722 inhabitants) (see social and demographic factors of the participants in Table 2).

### Primate Cultural Significance Index (PCSI)

The Primate Cultural Significance Index (PCSI) was composed of 11 sub-indices (cultural variables) influencing CS:${\text{PCSI}} = {\text{ OMF }}\left( {{\text{MED }} + {\text{ PET }} + {\text{ COM }} + {\text{ FOOD }} + {\text{ TOUR }} + {\text{ CSV}} + {\text{ EMO }} + {\text{ CONS }} + {\text{ ECO }} + {\text{ ABU}}} \right)$, where OMF = Order of mention frequency, MED = Medicinal significance, PET = Significance as companion animal, COM = Commercial significance, FOOD = Food significance, TOUR = Touristic significance, CSV = Significance in cosmovision, EMO = Emotional significance, CONS = Interest in conservation, ECO = Ecological significance, ABU = Perceived abundance.

In the PCSI, the values of each sub-index (except OMF) corresponds to the score average of the answers obtained from all the participants in each community for each species. The general index can reach a maximum value of 1000 (see scores in Table 1). The values for each sub-index ranged from zero (0) to ten (10). Some sub-indices were also composed of indicators, which were added, resulting in the total value of the sub-index. For these indicators, the maximum value of each sub-index was divided among the number of indicators that compose it, giving each indicator an equitable value. In turn, the possible answers of each indicator were scored giving higher values to the alternatives that represented greater importance (ordinal variable) (Table 1). The Order of Mention Frequency (OMF) [52] was modified from the Order of Mention (OM) proposed by Pieroni (2001) and Garibay-Orijel et al. (2007) [22, 51]. As this study had only two species, the OMF refers to the sum of the times (relative frequency) that each primate species was first mentioned spontaneously by the participants when asked about the primate species present in each community, divided by the total number of participants:

$${\text{OMF}} = \left( {{\text{No}}.\;{\text{of}}\;{\text{first}}\;{\text{mentions}}\;{\text{for}}\;{\text{each }}\;{\text{species/No}}.\;{\text{of}}\;{\text{paticipants}}} \right){ }10$$

**Table 1:** Values of sub-indices, indicators and possible answers in the PCSI

Sub-index	Indicator	Possible answers	Value
**MED** Medicinal significance (10) *nd* + *ef* + *ex*	*nd* Number of diseases that it cures (3.33)	Three or more	3.33
		Two	2.22
		One	1.11
		None	0
	*ef* Medicinal efficiency (3.33)	Very efficient	3.33
		Efficient	2.22
		Efficiency limited	1.11
		Not efficient	0
	*ex* Medicinal exclusivity (3.33)	Are just cured by this monkey	3.33
		There are other cures, but this species is better	2.22
		There are other species equally efficient to cure it	1.11
		There are better species more efficient to cure it	0
**PET** Significance as companion animal (10) *kee* + *des*	*kee* Keeping (5)	Has had it as pet	5
		Has not had it as pet	0
	*de* Desire (5)	Would love to have it as pet	5
		Would like to have it as pet	3.33
		Does not care about having it as pet	1.67
		Would never have it as pet	0
**COM** Commercial significance (10) *rv* + *hv*	*rv* Real price (5)	Is still sold	5
		Was sold before	2.5
		Has never been sold	0
	*hv* Hypothetical price (5)	Would sell it	5
		Would not sell it	0
**FOOD** Food significance (10) *con* + *fc* + *lc* + *tas*	*con* Consumption (2.5)	Has consumed it	2.5
		Has never consumed it	0
	*cf* Frequency of consumption (2.5)	Once a month	2.5
		Once a year	1.67
		A few times in life	0.83
		Never	0
	*lc* Last consumption (2.5)	Less than 5 years ago	2.5
		Between 5 and 30 years ago	1.67
		More than 30 years	0.83
		Never	0
	*tas* Taste (2.5)	Very tasty	2.5
		Tasty	1.67
		Regular	0.83
		Disgusting	0
**TOUR** Touristic significance (10)		Very attractive	10
		Attractive	6.67
		A little attractive	3.33
		Unattractive	0
**CSV** Significance in the Cosmovision (10)		More than 2 stories or beliefs	10
		2 stories or beliefs	6.67
		1 story or belief	3.33
		No story or belief	0
**EMO** Emotional significance (10) *see* + *hear*	*see* Emotion when sees them (9)	Positive	9
		None	0
		Negative	0
	*hear* Emotion when hears them (1)	Positive	1
		None	0
		Negative	0
**CONS** Interest in conservation (10)		Very high	10
		Medium	6.67
		Irrelevant	3.33
		Does not want that the species persists	0
**ECO** Ecological significance (10)		3 or more ecological functions	10
		2 ecological functions	6.67
		1 ecological function	3.33
		None	0
**ABU** Perceived abundance (10) *fs* + *gs*	*fs* Frequency of sightings (5)	Sees them every time that goes	5
		Sees them sometimes	3.33
		Almost never sees them	1.67
		Never sees them	0
	*gs* Group size (5)	More than 10 individuals	5
		More than 5	3.33
		More than 2	1.67
		Less than 2	0

In addition, the order of mention frequency value goes from 0 to 1, therefore it must be multiplied by 10 to adjust its values to the same scale as the other sub-indices [22].

In the PCSI, the OMF is multiplied by the other sub-indices to amplify the differences between the species [22] as several authors agree that the frequency and the order of mention are accurate variables to measure the cultural significance of species [23, 50]. This reduces biases and subjectivity [24]. Given that the PCSI is a composed additive index, the potential effect of each sub-index can be masked. We will discuss the performance of the sub-indices independently since compound indices should be a tool to separate, analyze and understand cultural significance phenomena, and also a technique to estimate it [22].

### Perceived significance of Los Tuxtlas Biosphere Reserve

The perceived significance of Los Tuxtlas Biosphere Reserve in the daily life of the inhabitants of each community was measured independently to the PCSI, on a 0–10 scale. The values for the answers were: Very important = 10, Important = 6.67, Less important = 3.33, Not important = 0.

### Data analysis

We compared the sub-indices of the PCSI between the communities and primate species using the U Mann–Whitney test. We evaluated the social and demographic factors of the participants (predictor variables: see social and demographic factors in Table 2) that influenced each sub-index (response variables were the sub-indices of cultural significance, Table 1: Medicinal, Companion animal, Commercial, Food, Touristic, Cosmovision, Emotional, Interest in conservation, Ecological, Perceived abundance) for spider and howler monkeys using Generalized Linear Models (GLMs) fit with gamma error distributions and inverse link (20 models). The OMF sub-index was not included in this portion of the analysis. To select the combination of predictor variables best fitting each dataset, we used the Akaike’s information criterion [53]. The model with the lowest ΔAIC score was considered the best-fitting model. However, in cases where the next best-fitting model was within two AIC points, indicating a plausible alternative, we selected the most parsimonious model (i.e., the model with the fewest predictors) [53]. With this information we calculated the Akaike weight (*wi*) for each model, which ranged from 0 to 1 and was interpreted as the relative probability that a model is the best among all those evaluated. This means that models with higher *wi* values will be the most probable and will have a greater explanatory power for the response factors. For the selected models, we calculated the relation between variables extracting the P value and slope from the GLM test. The GLMs were performed with the *nlme* package [54] and for fitting maximum likelihood models we used *bbmle* package [55] within the statistical program R (version 3.2.0) [56].

**Table 2:** Social and demographic factors and percentage of participants within and outside the TBR

Social/demographic factor	Levels	%People within the TBR	% People outside the TBR
TBR location	Inside	100	
	Outside		100
Gender	Male	46	48
	Female	54	52
Age	Young (< 35 years)	49	21
	Adult (35–59)	40	43
	Elder (> 60)	11	36
Origin	Native	85	99
	Foreign	15	1
Perceived language proficiency	Perfect	49	11
	Good	9	22
	Limited	25	41
	None	17	26
Education level	Bachelor	2	0
	Post-secondary	15	12
	Secondary	35	18
	Primary	48	59
	None	0	11
Occupation	Farmer	46	47
	Home duties	40	44
	Student	7	4
	Other	5	3
	None	2	1
Religion	Catholic	4	43
	Protestant	44	18
	None	52	39

Total of participants in the community within the TBR (Piedra Labrada; *N* = 81) and outside (Los Mangos; *N* = 91)

## Results

### Ethnographic information

Primates do not appear in the myths or legends of Popolucas. However, there are a number of popular beliefs associated with both howler and spider monkeys. Howler monkeys are believed to kidnap and sexually abuse women and spider monkeys are related to supernatural beings. Nevertheless, both species are associated with joy and fun. The howler monkeys are widely considered to provide a meteorological service by predicting the arrival of rainy or dry seasons when howling. Petroglyphs created by the ancient Olmecs with figures of monkeys were found in the Piedra Labrada community. The Popolucas do not traditionally consume primates as food, but they did use spider monkeys medicinally and, in the past, used to keep the young of this species as companion animals to later release them. On an economic level, spider monkeys have been extracted for consumption or to be used as bait for fishing by non-indigenous people in the area. Popolucas in the communities of study consider that the populations of both species have decreased due to deforestation and hunting. Additionally, they perceive the loss of natural vegetation due to cattle ranching, commercial agriculture and timber extraction as the main threats to primates. They view the sale of traditional indigenous lands to non-indigenous settlers as an indirect threat. Finally, the Popolucas of these communities express a willingness to conserve primates so that young people and future generations may know them. They consider that tourism is a worthwhile strategy to aid non-human primate conservation, but to offer tourism services, they will need external support. For detailed ethnographic information about cultural interactions of Popolucas with primates see Pinto-Marroquin & Serio-Silva (2020) [49].

### Primate Cultural Significance Index (PCSI)

The results of the PCSI (Table 3) showed that spider monkeys play a more important role in the Popoluca community inside the TBR (S-PCSI = 151.9) rather than outside the TBR (S-PCSI = 60.9), which supports our hypothesis. On the other hand, the lower PCSI value for howler monkeys inside (H-PCSI = 209.5) rather than outside the reserve (H-PCSI = 266.1) does not support our hypothesis. For both species in both communities, the sub-indices with higher values were interest in conservation (CONS) and touristic significance (TOUR). The significantly higher sub-indices values in the community inside rather than outside the TBR, for both species, were interest in conservation (CONS), touristic significance (TOUR) and perceived abundance (ABU). Inside the TBR, for spider monkeys, the higher values were the significance as companion animal (PET) and emotional significance (EMO). For the same species, but in the community outside the TBR, the higher values were for medicinal significance (MED) and food significance (FOOD).

**Table 3:** Results and comparisons of the PCSI for both primate species between communities

	Index results				Comparisons	
Index, sub-index, *indicator*	Piedra Labrada		Los Mangos		Community location Within vs outside	
	Within TBR		Outside TBR			
	*A. geoffroyi*	*A. palliata*	*A. geoffroyi*	*A. palliata*	*A. geoffroyi*	*A. palliata*
**PCSI**	**151.98**	**209.55**	**60.99**	**266.13**		
**OMF**	**4.07**	**5.56**	**1.89**	**7.89**		
**MED**	**0.29**	**0.09**	**3.29**	**0.01**	***	**ns**
*nd*	0.25	0.01	0.67	0.01	***	ns
*ef*	0.01	0.04	1.60	0.00	***	ns
*ex*	0.03	0.04	1.02	0.00	***	ns
**PET**	**2.82**	**0.82**	**1.83**	**1.00**	*	**ns**
*kee*	0.99	0.12	0.61	0.00	ns	ns
*de*	1.83	0.70	1.22	1.00	*	ns
**COM**	**0.86**	**0.34**	**0.8**	**0.33**	**ns**	**ns**
*rp*	0.49	0.15	0.58	0.11	ns	ns
*hp*	0.37	0.19	0.22	0.22	ns	ns
**FOOD**	**1.84**	**0.24**	**3.24**	**0.17**	**	**ns**
*con*	0.62	0.12	1.22	0.06	**	ns
*fc*	0.31	0.03	0.71	0.02	**	ns
*lc*	0.32	0.03	0.45	0.03	**	ns
*tas*	0.59	0.06	0.86	0.06	ns	ns
**TOUR**	**9.22**	**9.22**	**8.08**	**8.19**	**	**
**CSV**	**0.33**	**3.78**	**0.22**	**4.33**	**ns**	**ns**
**EMO**	**6.33**	**6.26**	**3.00**	**5.09**	***	**ns**
**CONS**	**9.55**	**9.42**	**8.66**	**8.52**	**	**
**ECO**	**1.69**	**1.69**	**1.74**	**1.78**	**ns**	**ns**
**ABU**	**3.89**	**5.84**	**1.41**	**4.33**	***	***
*fs*	1.89	3.04	0.52	2.61	***	ns
*gs*	2.00	2.80	0.89	1.72	***	***

*P* < 0.05 = *, *P* < 0.01 = **, *P* < 0.001 = *** and *ns* no significative difference. Sub-indices are in bold and indicators in low case and italics. *PCSI* Primates Cultural Significance Index; *OMF* Order of mention frequency; *MED* Medicinal, *nd* number of diseases that it cures, *ef* efficiency, *ex* exclusivity; *PET* Companion animal, *kee* keeping, *de* desire; *COM* Commercial, *rp* real price, *hp* hypothetical price; *FOOD* Food, *con* consumption, *fc* frequency of consumption, *lc* last consumption, *tas* taste; *TOUR* Touristic; *CSV* Cosmovision; *EMO* Emotional; *CONS* Conservation; *ECO* Ecological; *AUB* Perceived abundance, *fs* frequency of sightings, *gs* group size

In general, the sub-indices with the lowest value were commercial significance (COM) and ecological significance (ECO). Howler monkeys showed a greater value than spider monkeys in the overall result of the PCSI. The sub-indices comparison between species in both communities indicated that howler monkeys had higher significance in the cosmovision (CSV) and perceived abundance (ABU), and lower medicinal (MED) and food (FOOD) significance; whereas spider monkeys showed lower significance in cosmovision (CSV).

### Perceived significance of Los Tuxtlas Biosphere Reserve (TBR-P)

Significant differences were found in the perceived significance of TBR between the community inside (Piedra Labrada, TBR-P = 6.91) and outside the reserve (Los Mangos, TBR-P = 5.11) (*u* = 2556, *p* = 0.0004, *n* = 171). Most participants inside the TBR considered the reserve as important in people’s daily life (80%). In the community outside the TBR, half of the participants recognized the reserve as important, while the remaining considered it as not important (28%) or ignored the existence of the reserve (22%). In both communities the participants acknowledged the reserve function for conservation of wild animals and forest, water supply, climate, and air quality regulation; as well as its role in environmental education.

### Social and demographic factors influencing cultural significance of primates

The results of GLMs (Table 4) showed that social and demographic factors influenced nine sub-indices of cultural importance out of 10 tested per species; likewise, 15 models were significant out of 20 tested (six for spider monkeys; nine for howler monkeys). In spider monkeys the sub-indices (see names, abbreviations and calculations in Table 1) that were explained by social and demographic factors were: FOOD, TOUR, EMO, ICON, ECO, ABU; while for howler monkeys were: MED, PET, FOOD, TOUR, CSV, EMO, ICON, ECO AND ABU.

**Table 4:** Results of GLMs for social and demographic factors that influenced the sub-indices of the PCSI

Response variable	Primate species	Social/demogr. factors	Deviance	Pr(> Chi)	*df*	Factors levels					Explanation power		
						Contrasts	Estimate	*t* value	*p*	*df*	AIC	ΔAIC	*wi*
MED	*A. geoffroyi*	TBR location	30.47	***	1	Inside:Outside	0.47	8.51	***	167	659.7	3	0.18
		Age	17.14	***	2	Young:Elder	0.33	4.31	***	167			
						Adult:Young	0.24	3.20	***	167			
						Adult:Elder	− 0.08	− 2.13	*	167			
	*A. palliata*	Gender	0.40	*	1	Female:Male	− 0.21	− 2.17	*	163	**446.2**	**0**	**0.99**
		Occupation	3.69	***	4	Farmer:Student	− 0.46	− 7.14	***	163			
		Language	0.55	*	3	Perfect:Limited	0.01	2.53	*	163			
		Gender: Occupation	0.53	*	3	FemaleFarmer:MaleStudent	− 0.49	− 2.43	*	163			
PET	*A. geoffroyi*	TBR location	3.8	*	1	Inside:Outside	0.09	2.16	*	169	742.0	0.8	0.29
	*A. palliata*	Occupation	0.3	*	4	Farmer:Student	− 0.01	− 2.36	*	163	**647.6**	**0**	**1**
						Student:Home	− 0.01	− 2.79	**	163			
						Student:Other	− 0.02	− 2.82	**	163			
		Language	0.2	*	3	Good:Perfect	0.01	2.18	*	163			
						Perfect:Limited	0.01	2.51	*	163			
COM	*A. geoffroyi*	ns	ns	ns	ns	ns	ns	ns	ns	ns	ns	ns	ns
	*A. palliata*	ns	ns	ns	ns	ns	ns	ns	ns	ns	ns	ns	ns
FOOD	*A. geoffroyi*	TBR location	0.54	**	1	Inside:Outside	− 0.01	− 2.36	*	161	**842.8**	**0**	**0.58**
		Gender	0.32	*	1	Female:Male	− 0.01	− 2.11	*	161			
		Language	0.92	***	3	Good:None	0.01	2.87	**	161			
						None:Perfect	0.01	2.30	*	161			
						None:Limited	0.01	3.00	**	161			
		Education	0.52	*	4	Bachelor:PostSecund	0.03	2.36	*	161			
						Bachelor:Secundary	0.03	2.28	*	161			
	*A. palliata*	Gender	0.06	*	1	Female:Male	0.00	2.39	*	169	**445.6**	**0**	**0.99**
TOUR	*A. geoffroyi*	TBR location	0.60	***	1	Inside:Outside	− 0.01	− 2.60	*	169	**842.8**	**0**	**0.58**
	*A. palliata*	TBR location	0.48	*	1	Inside:Outside	− 0.11	− 2.42	*	169	**974.7**	**0**	**1**
CSV	*A. geoffroyi*	ns	ns	ns	ns	ns	ns	ns	ns	ns	ns	ns	ns
	*A. palliata*	TBR location	0.49	0.07	1	Inside:Outside	0.03	2.29	*	167	**753.6**	**0**	**0.7**
		Religion	1.12	*	2	Protestant:None	− 0.04	− 2.65	**	167			
EMO	*A. geoffroyi*	TBR location	15.59	***	1	Inside:Outside	0.11	4.40	***	165	**291.6**	**0**	**1**
	*A. palliata*	Gender	7.99	***	1	Female:Male	− 0.06	− 3.45	***	165	**982.4**	**0**	**0.62**
		Origin	2.41	*	1	Foreign-Native							
		Language	5.40	*	3	Good:None	− 0.07	− 2.27	*	165			
						Good:Perfect	− 0.07	− 2.27	*	165			
CONS	*A. geoffroyi*	TBR location	0.33	**	1	Inside:Outside	0.01	2.76	**	168	**829.3**	**0**	**1**
		Gender	0.21	*	1	Female:Male	− 0.01	− 2.36	*	168			
	*A. palliata*	TBR location	34.92	**	1	Inside:Outside	− 1.09	− 3.14	**	167	**757.1**	**0**	**1**
		Origin	19.89	*	1	Foreign:Native	1.33	2.04	*	168			
ECO	*A. geoffroyi*	Gender	7.32	***	1	Female:Male	− 0.16	− 3.79	***	169	**638.0**	**0**	**1**
	*A. palliata*	Gender	7.74	***	1	Female:Male	− 0.16	− 3.91	***	169	**636.5**	**0**	**1**
ABU	*A. geoffroyi*	TBR location	20.94	***	1	Inside:Outside	0.20	5.13	***	169	**740.5**	**0**	**0.54**
		Gender	2.83	*	1	Female:Male	− 0.07	− 2.06	*	169			
	*A. palliata*	TBR location	97.06	***	1	Inside:Outside	− 1.42	− 3.80	***	168	**781.0**	**0**	**1**
		Gender	189.71	***	1	Female:Male	2.11	5.93	***	168			
		Education	58.34	*	4	Bachelor:None	4.09	2.23	*	165			
						Bachelor:Secundary	3.95	2.35	*	165			

Significative results in bold. *P* < 0.05 = *, *P* < 0.01 = **, *P* < 0.001 = ***, *df* degrees of freedom, *MED* Medicinal, *PET* Companion animal, *COM* Commercial, *FOOD* Food, *TOUR* Touristic; *CSV* Cosmovision; *EMO* Emotional; *CONS* Conservation; *ECO* Ecological; *AUB* Perceived abundance. Response variable = sub-index. Spider monkey (*A. geoffroyi*) and howler monkey (*A. Palliata*). *AIC* Akaike’s information criterion, *ΔAIC* difference in AIC compared to the best model, *wj* Akaike weight. Note that GLMs were fit with gamma error distributions and inverse link

The social and demographic factors that most explained each sub-index of cultural significance for both primate species were the location of communities with respect to the TBR (TBR location; nine sub-indices out of 20 proved), gender (nine sub-indices out of 20 proved). The TBR location factor in spider monkeys influenced the sub-indices FOOD, TOUR, EMO, CONS and ABU; while for howler monkeys influenced TOUR, CSV, CONS and ABU. The gender factor in spider monkeys influenced FOOD, CONS, ECO and ABU; while in howler monkeys influenced MED, FOOD, EMO, ECO and ABU. Detailed results of social and demographic factors influencing the sub-indices are presented in Table 4.

The results of both tests (U Mann–Whitney and GLMs) regarding the influence of Los Tuxtlas Biosphere Reserve on all the sub-indices were consistent with the exception of significance in cosmovision (CSV) for howler monkeys, which was only found in the GLMs.

## Discussion

The Primate Cultural Significance Index (PCSI) showed differential cultural relevance for both primate species between Popolucan communities within (Piedra Labrada) and outside (Los Mangos) the Los Tuxtlas Biosphere Reserve (TBR). We found that spider monkeys (*A. geoffroyi*) were culturally significant inside, while howler monkeys (*A. palliata*) were viewed as culturally significant outside of the reserve, which partially supports our hypothesis.

**Fig. 2:**
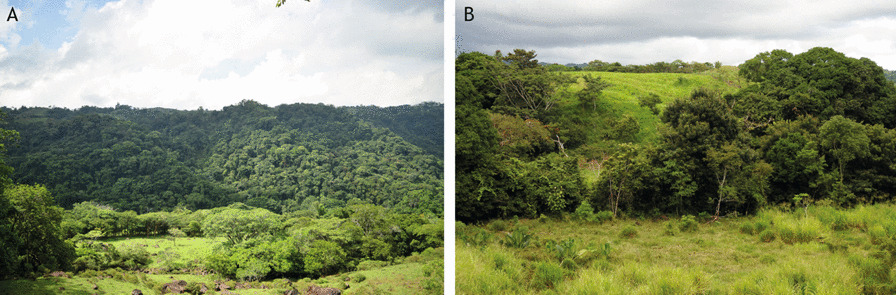
Type of surrounding habitat of the study communities. **A** inside the TBR (Piedra Labrada). **B** outside the TBR (Los Mangos). (Photograph A by Jorge Ramos, photograph B by the first author)

The PCSI was strongly influenced by the order of mention frequency (OMF). It has been argued that most mentioned species are often the more abundant or more likely to be seen [24]. When comparing the OMF between communities, the spider monkeys were mentioned more frequently inside the reserve. This may be related to a major abundance due to the fact that their habitat is more connected and conserved within the reserve [39, 57] (Fig. 2), also explaining the higher perceived abundance (ABU) and emotional significance (EMO) for this species. Conversely, the howler monkeys had higher OMF and EMO values outside the reserve, even when their perceived abundance (ABU) was higher inside the reserve. This may be explained by howler monkeys being the only primate recognized as present outside the reserve in the majority of surveys.

“Spider monkeys no longer exist around here. The last time they were seen was around the 70's. The howler monkeys are the only ones present here in Los Mangos”. F. Pascual, Los Mangos, July 2016.

Spider monkeys are rarely seen outside the reserve as a result of forest loss and fragmentation that have limited their food resources making this species more vulnerable [57]. Primate demographic studies in Los Tuxtlas reported that howler monkeys are able to survive in more disturbed habitats with small forest fragments including anthropogenic habitats [58]. In contrast, spider monkeys require forests with continuous coverage and more tree diversity [59, 60].

The sub-indices with greater cultural significance inside the reserve for both species were perceived abundance (ABU), interest in conservation (CONS) and touristic significance (TOUR). Primate abundance within Los Tuxtlas Biosphere Reserve (TBR) has remained relatively stable since the 1980s [57, 60] and the forest cover increased by 2402.97 ha (1.55% of TBR area) between 2006 and 2016 [39]. This is probably due to the protected area regulations which ban logging and hunting [61]. The interest in conservation (CONS) and the touristic significance of primates (TOUR), in addition to being more important inside the TBR, were the highest sub-indices for both communities and primate species. It has been estimated that in Los Tuxtlas region, since the Spanish colonization, the population of primates has decreased by 80% with some groups persisting in fragmented and isolated vegetation patches [59, 60]. Spider monkey populations have decreased faster than howler monkeys [60]. This situation, along with the influence of the reserve and of primate conservation education strategies in the region, such as the annual festival *Changos y monos tesoros de los Tuxtlas* which has been held since 2014, may have motivated people to conserve primates, particularly in communities within the reserve.

“If someone is hunting primates here, we go and report it to the municipal agent. We want these animals to continue to exist so that the children will get to know them”. A. Albino, Piedra Labrada, October 2016.

In the neighboring locality of Catemaco, the economic income generated by tour boats to observe exotic primates (*Macaca arctoides*) and spider monkeys in the Catemaco lake is more important than other economic activities such as agriculture, livestock farming and working in protected natural areas [62]. This case was mentioned by the participants during this study as an example of how the presence of primates represents an income opportunity.

“In Catemaco many tourists go to see the monkeys, which leaves economic profit to the inhabitants. If tourism here was organized in such a way that visitors would come to see the monkeys by the river, it would be a good economic opportunity for us”. J. González, Los Mangos, July 2016.

Since the 1980s, tourism has been increasingly explored as a way to conserve primates around the globe, which in consequence protects their habitat and generates an income for local people [63]. For example, in the Central African Republic, ecotourism has been established with the main attraction of observing groups of gorillas in the wild, economically benefiting the local population and the primate populations themselves [64]. In some temples in Southeast Asia, monkeys are an important attraction for visitors; local human communities receive economic benefits from tourism and primates are perceived as culturally important, receiving protection by being associated with sacred sites [7, 65]. Tourism has also been implemented as a strategy in the Colombian Amazon in Tikuna territories where conservation centers have been established. There, tourists can see rescued monkeys and in doing so, contribute to the local economy and benefits people [12].

For spider monkeys, we found higher cultural significance within the reserve regarding emotional significance (EMO) and significance as companion animal (PET). This result may be related to positive emotions associated with a higher possibility of sighting them (greater perceived abundance ABU), a higher desire that this species continues to exist (CONS), and Popolucas’ ancestral tradition that encourages allowing spider monkeys to roam free around their houses.

“A long time ago in my family, we had three young spider monkeys. Once they were tamed, we left them loose around the house and when they were older, they returned to the forest” A. Albino, Piedra Labrada, October 2016.

Since the language proficiency level is an indicator of cultural strength [66] and there is higher perceived language proficiency in the community within the TBR (Table 2), Popolucan ancestral traditions, such as this one, even when they are banned, will continue. In 2003 in Mexico, most primates kept as pets (70%) corresponded to spider monkeys [13] and in Mesoamerica they have been commonly kept as pets because they are more tamable and versatile, while howlers are difficult to tame [16, 19]. The appropriation of monkeys as companion animals is a common practice in Latin America, an example of this is documented for the Maijuna of the Ecuadorian Amazon [10], among Yanomami groups from Brazil and Venezuela [9] and among Lokono, Kari'na and Warao groups in Guyana [14] where, commonly, people “adopt” infants after the mothers are hunted.

Conversely, the higher sub-indices for spider monkeys outside the TBR were related to their extraction (food significance FOOD and medicinal significance MED) and may be explained by the fact that law enforcement in the reserve prevents wildlife hunting [36]. However, traditionally, Popolucas do not eat spider monkeys despite the fact that they describe the meat as edible (Table 3) while, in other traditional societies, the genus *Ateles spp*. is the most appreciated primate for human consumption [19].

"None of us Popolucas eat monkeys. We were told that it is not good to eat them because they are people... We have heard that spider monkey meat tastes good". J. Albino, Piedra Labrada, October 2016.

Moreover, there is a mestizo tradition of consuming spider monkeys in the nearby municipality of Catemaco, where their meat is highly valued, influencing their use as food in Los Mangos. Some cases have been documented in which people do not consume primate meat (for example in certain areas of the Brazilian Amazon and on the border between Colombia and Venezuela) because they perceive these organisms as similar to human children [19, 67, 68], however the consumption of meat from different species of monkeys is common among indigenous cultures throughout the Neotropics. The consumption of various primate species has been reported among the Lacandones of Chiapas, Mexico [24]; among the Maijuna of the Peruvian Amazon [10]; among the Waoriani of the Ecuadorian Amazon [69]; among the Tikuna in Colombia [70], and among the Tacana of Bolivia [15], just to mention a few examples. Meat is regularly boiled, roasted or smoked for consumption and is generally perceived as excellent tasting meat, even preferred over other wild game [10, 14, 15]. Among the Tikuna of southeastern Colombia, the wooly monkey is consumed as a ritual meal [12]. On other occasions, the meat is not destined for household-consumption but is brought to local markets to be sold as bushmeat, where such sale can generate cash to satisfy other household needs.

We also found a higher medicinal significance (MED) outside the reserve for the spider monkey probably because this species is considered by study participants as the only cure for many diseases. The use of monkeys in traditional medicine is also a widespread practice throughout the world, where the use of more than 100 species of primates in ethnomedical practices or magical-religious rituals has been recorded [11, 71]. In Latin America, for example, the use of howler throat sacs has been recorded to cure laryngitis among the Tikunas [12] and the use of howler hairs to counter scorpion stinging and to ward off evil spirits among different groups in Guyana has also been documented [14].

For howler monkeys we found that their significance in cosmovision (CSV) was influenced by the participants outside the reserve, where the Popolucan cultural influence is not as strong. Also, by people belonging to protestant churches, suggesting that reported stories and beliefs are not ancestrally popolucans. Since the 1960’s, the number of Protestants churches in Mexico has grown exponentially [72], fostering new traditions [73] and focusing particularly on reported stories by women [74]. In this study, many women narrated that howler monkeys chase and try to kidnap them (30% of participants). Legends relating to women and primates extend beyond howler monkey species in other cultures. Among the Takana of the Bolivian Amazon, it is commonly said that spider monkeys can kidnap women from villages and take them to the jungle [15]. Participants widely conveyed that howler monkeys can predict and announce weather variations when they vocalize (80%). This ethnobiological knowledge has been described throughout the geographic range of this genera, for example in Argentina by the Qom (Toba) People of the Gran Chaco and in Guyana by the Lokono, Kari’na, and Warao Peoples [14, 75].

“When the weather's going to change, the monkeys start screaming. They announce when it is going to rain, when the sun is going to warm, when it is going to hit a *sur* (dry season) or a *norte* (rainy and cold season)”. V. Lázaro, Los Mangos, July 2016.

The demographic factor that most influenced the cultural significance (sub-indices, Table 4) of primates after the TBR location was gender. Males in our study especially influenced the perceived abundance and emotional significance for both species, and the interest in conservation for spider monkeys. Men also had influence in the food significance of spider monkeys in the community outside the reserve. This may be explained by the fact that men do crop work near the forest, increasing their interactions with primates, while women are mainly in charge of domestic tasks in their houses, sometimes helping in the fields. Traditionally, Popolucan adult men are the ones who access the forest to hunt and fish [76]. We also found a higher ecological knowledge (ECO) among men compared to that of women. This pattern has been observed in other countries in the region such as in Costa Rica where it was reported that although women visit the forest as often as men, they may show less knowledge about ecology and conservation [77], which, in turn, can affect their attitudes and values towards the environment [78]. In southern Mexico, a study has shown that when educational programs promoting primate conservation are offered equally between boys and girls, gender differences in ecological knowledge about monkeys disappear [79]. Native participants influenced higher interest in conservation for howler monkeys and emotional significance for both species, which reflects a sense of ownership, and a greater concern and appreciation of their local biodiversity than immigrants from other areas.

The differences we found between communities can be attributed to the conservation and education programs implemented by the reserve managers. These differences, however may also be due to uneven socioeconomic development which is, in part a consequence of government and NGO investment in and promotion of sustainable economic development inside the boundaries of the reserve [36]. In addition, we found a higher perception of the importance of the protected area (TBR) by participants within the reserve rather than outside the reserve, as expected. Studies in Asia and Africa also have reported that people living closer to natural parks perceived more benefits and had a positive attitude towards conservation and the protected areas [42, 80]. Likewise, at various locations across Latin America, conservation actions undertaken within protected areas has led to a decrease in hunting and a reduction in the consumption of primates for food or traditional remedies [12, 70, 81] thereby contributing to the recovery of primate populations.

## Conclusion

Spider monkeys play a more culturally significant role in the Popoluca community inside the reserve (Piedra Labrada) when compared to the community outside (Los Mangos). Howler monkeys are more culturally important outside the TBR likely because they are the only species recognized as currently present in this community. The sub-indices with greater CS inside the reserve for both species were mainly related to conservation, while the indices that were more significant outside of the reserve were related to extraction and consumption of spider monkeys. The social groups that perceived a higher CS of primates were men and interview participants living inside the reserve. The main differences in CS of primates between communities may be tied to the conservation and sustainable development programs carried out inside the reserve and which likely influence attitudes towards primates inside the reserve. The Popolucan ancestral traditions are more preserved within the community inside the reserve, and thus also likely has an important effect on the differing CS of primates between the two study communities.

The high interest in conservation and ecotourism in both communities represents a strategic opportunity for primate conservation in Los Tuxtlas region. Therefore, conservation efforts should encourage people to remain on their ancestral lands, by supporting sustainable management and economic opportunities for communities. Such programs could follow the pattern of other successful conservation programs which are considered to be long term, small-scale, and relatively low-budget [82]. To implement responsible tourism programs, it is essential to consider that tourists can expose primates to physiological stress and anxiety, producing changes in their behavioral patterns and also exposing humans and primates alike to injury and disease [83]. Tourism activities should be implemented promoting conservation education, avoiding direct interactions, and minimizing impacts on the ecosystems. Traditional values of indigenous people must also be considered to avoid conflicts of interest, project failures, and negative effects on their culture [84]. In order to ensure long-term sustainability for ecotourism, community participation and the generation of profits are essential [85]. Primate conservation, education and ecotourism programs in Los Tuxtlas should be gender inclusive and focus on strengthening ecological knowledge and cultural traditions in the social groups less interested about conservation such as people outside the reserve, women, and non-natives. These programs should also focus on taking advantage of the ecological knowledge and interest in conservation of native men inside the reserve for leadership.

The conservation of non-human primates is an objective that requires theoretical and methodological frameworks that go beyond the dictates of disciplines such as Conservation Biology [86]. From the humanities, epistemic concepts such as “post humanism” have been proposed as an attempt to include non-human beings in the social sciences and humanities [87, 88], or the so-called “multispecies ethnographies”, that pose a blurring of the ontological line between human and nature [89]. For Robinson and Remis (2018) [86], multispecies ethnography can foster a more holistic and transdisciplinary research, and a framework that allows highlighting the wide and intricate networks in which primates, humans and non-humans, as well as other organisms coexist and interact. Other perspectives such as Evolutionary Ethnobiology theorize about how the changes generated by humans to the environment influence the evolutionary process of other organisms, giving feedback in a dialectical way to the evolution of humans [90].

Ethnoprimatologists have been exploring holistic paths, for example promoting a common field of intersection between anthropology and primatology for biological and cultural conservation [7, 91]; and also recognizing the blurring boundaries between primates and humans through the deep understanding of culturally complex relationships among indigenous peoples [17, 92]. There are also multiple examples of current transdisciplinary research in ethnoprimatology that go from collaborative research among anthropologists, biologists, and indigenous peoples [9], the interpretation of ancestral codes, values, and links in the collective unconscious of people for conservation [93] to the understanding of primates as “other-than human persons” from a cognitive point of view [94] or from a multispecies perspective [89]. Finally, as we find in this study, quantitative ethnoprimatology, particularly cultural significance indexes, provides an analytical framework for synthesizing information and patterns across socio-ecological systems. The cultural significance index highlights the elements that people consider most important in their relationship with primates and thereby can facilitate the design of culturally and socially relevant conservation strategies, strategies that are more likely to be effective once implemented.

## Supplementary Information

**Additional file 1**. Cultural significance of primates’ questionnaire. Applied to the participants of the community inside (Piedra Labrada, *N* = 81) and the community outside (Los Mangos, *N* = 91) of Los Tuxtlas Biosphere Reserve (Additional file 1).

## Data Availability

The datasets used and analyzed during the current study are available from the corresponding author on reasonable request.

## Abbreviations

ABU: Perceived abundance
AIC: Akaike information criterion
CFSI: Cultural Food Significance Index
COM: Commercial significance
CONANP: Comisión Nacional de Áreas Naturales Protegidas
CONS: Interest in conservation
CS: Cultural significance
CSI: Cultural Significance Index
CSV: Significance in cosmovision
ECO: Ecological significance
EMCSI: Edible Mushrooms Cultural Significance Index
EMO: Emotional significance
FOOD: Food significance
GLM: Generalized linear model
IMSS: Instituto Mexicano del Seguro Social
INPI: Instituto Nacional de los Pueblos Indígenas
MED: Medicinal significance
OMF: Order of mention frequency
PET: Significance as companion animal
PCSI: Primate Cultural Significance Index
TBR: Los Tuxtlas Biosphere Reserve
TOUR: Touristic significance
UNESCO: United Nations Educational, Scientific and Cultural Organization
