# Supplementary material for: The Primate Cultural Significance Index: applications with Popoluca Indigenous people at Los Tuxtlas Biosphere Reserve
Source: J Ethnobiol Ethnomed. 2021 Oct 9;17:57. doi: 10.1186/s13002-021-00483-8 (PMC8502360; doi:10.1186/s13002-021-00483-8)
Supplement: Supplementary file 1 — Additional file 1. Cultural significance of primates’ questionnaire. Applied to the participants of the community inside (Piedra Labrada, N = 81) and the community outside (Los Mangos, N = 91) of Los Tuxtlas Biosphere Reserve (Additional file 1). [file 13002_2021_483_MOESM1_ESM.pdf]

CULTURAL SIGNIFICANCE OF PRIMATES QUESTIONNAIRE

Date:________________________________ Community:____________________________________

PERSONAL INFORMATION

Full name:			Age:
Gender: F☐ M☐	Occupation:		
Place of origin:		Time living in the community:	
Popoluca language proficiency: Perfect☐ Good☐ Limited☐ None ☐			
Religion:		Education level:	

**1**	What are the types of monkeys present here, in the community?<br>(Memo: *write "1" to the first mentioned and "2" to the second*)	Spider monkey ☐	Howler monkey ☐

Corroborate the species identity by showing the pictures of both species
(Memo: *ask all the following questions for both species even if they were not mentioned in question 1*)

	**CHANGO or SPIDER MONKEY (SM)**	**MONO ZAMBO or HOWLER MONKEY (HM)**
2	SM have caused you damage or loss? Yes☐ No☐	HM have caused you damage or loss? Yes☐ No☐
3	What are the diseases that can be cured with the SM?<br>A. ______________________None (*go to 6*)<br>B. ______________________One<br>C. ______________________Two<br>D. ______________________Three or more	What are the diseases that can be cured with the HM?<br>A. ______________________None (*go to 6*)<br>B. ______________________One<br>C. ______________________Two<br>D. ______________________Three or more
4	How good does the SM works to cure those diseases?<br>A. Very good<br>B. Good<br>C. Regular<br>D. Does not work	How good does the HM works to cure those diseases?<br>A. Very good<br>B. Good<br>C. Regular<br>D. Does not work
5	The diseases mentioned:<br>A. They are only cured by the SM<br>B. There are other cures but the SM is the best<br>C. There are other cures just as good as the SM	The diseases mentioned:<br>A. They are only cured by the HM<br>B. There are other cures but the HM is the best<br>C. There are other cures just as good as the HM
6	Have you had SM as pets? Yes☐ No☐	Have you had HM as pets? Yes☐ No☐
7	If it were not forbidden ¿would you like to have a SM as a pet?<br>A. I would love to<br>B. I would like to<br>C. It does not matter to me<br>D. I would not like to	If it were not forbidden ¿would you like to have a HM as a pet?<br>A. I would love to<br>B. I would like to<br>C. It does not matter to me<br>D. I would not like to
8	The spider monkey:<br>A. Is still sold<br>B. Was sold before<br>C. Has never been sold	The howler monkey:<br>A. Is still sold<br>B. Was sold before<br>C. Has never been sold
9	If it weren't forbidden and you could sell a SM, how much would you sell it for?<br>A. Price________________________________<br>B. Wouldn't sell it	If it weren't forbidden and you could sell a HM, how much would you sell it for?<br>A. Price________________________________<br>B. Wouldn't sell it
10	Have you eaten SM? Yes☐ No☐ (go to 13)	Have you eaten HM? Yes☐ No☐ (go to 13)
11	How often did you eat SM?<br>A. Once a month or more<br>B. Once a year<br>C. More than once in life<br>D. Only once in a lifetime	How often did you eat HM?<br>A. Once a month or more<br>B. Once a year<br>C. More than once in life<br>D. Only once in a lifetime
12	How long has it been since the last time you ate SM?<br>A. Less than 5 years<br>B. Between 5 and 10 years<br>C. Between 10 and 30 years<br>D. More than 30 years	How long has it been since the last time you ate HM?<br>A. Less than 5 years<br>B. Between 5 and 10 years<br>C. Between 10 and 30 years<br>D. More than 30 years
13	How tasty the SM is said to be?<br>A. Very tasty<br>B. Tasty	How tasty the HM is said to be?<br>A. Very tasty<br>B. Tasty

	C. Regular<br>D. Disgusting	C. Regular<br>D. Disgusting
14	Do you think SM can be a tourist attraction?<br>A. A lot<br>B. More or less<br>C. A little<br>D. I do not think so	Do you think SM can be a tourist attraction?<br>A. A lot<br>B. More or less<br>C. A little<br>D. I do not think so
15	How many tales or stories about SM do you know?<br>A. None<br>B. ______________________________1<br>C. ______________________________2<br>D. ______________________________3<br>E. ______________________________> 3	How many tales or stories about HM do you know?<br>A. None<br>B. ______________________________1<br>C. ______________________________2<br>D. ______________________________3<br>E. ______________________________> 3
16		Have you heard that monkeys announce something when they howl?<br>A. ______________________________<br>B. ______________________________<br>C. ______________________________<br>D. ______________________________
17	Have you seen the SM? ____ When you see it, what do you feel?<br>A. Joy<br>B. Nothing<br>C. Fear<br>D. Other________________	Have you seen the HM? ____ When you see it, what do you feel?<br>A. Joy<br>B. Nothing<br>C. Fear<br>D. Other________________
		What do you feel when you hear a HM howling??<br>A. Joy<br>B. Nothing<br>C. Fear<br>D. Other________________
18	Would you like SM to continue to exist?<br>A. I would like it very much<br>B. I would like it<br>C. I do not care<br>D. I would not like it	Would you like HM to continue to exist?<br>A. I would like it very much<br>B. I would like it<br>C. I do not care<br>D. I would not like it
19	Why are SM important in the forest? (Ecological functions)<br>A. Not important or don't know<br>B. ____________________ one function<br>C. ____________________ two functions<br>D. __________________ ≥Three functions	Why are HM important in the forest? (Ecological functions)<br>A. Not important or don't know<br>B. ____________________ one function<br>C. ____________________ two functions<br>D. __________________ ≥Three functions
20	Of the times you go to the forest, how often do you see the SM?<br>A. Every time you go<br>B. Almost always<br>C. Sometimes<br>D. Almost never<br>E. Never<br>F. Never go to the forest	Of the times you go to the forest, how often do you see the HM?<br>A. Every time you go<br>B. Almost always<br>C. Sometimes<br>D. Almost never<br>E. Never<br>F. Never go to the forest
21	How many SM in a group do you use to sight when you find them?<br>A. One<br>B. Between 2 and 5<br>C. Between 6 and 10<br>D. More than 10	How many HM in a group do you use to sight when you find them?<br>A. One<br>B. Between 2 and 5<br>C. Between 6 and 10<br>D. More than 10

INFLUENCE OF LOS TUXTLAS BIOSPHERE RESERVE

22. Is Los Tuxtlas Biosphere Reserve important to you in your daily life?

A. A lot
B. More or less
C. Not at all
D. It affects me negatively
Why?____
